# Single-Port Percutaneous Nephroscopy combined with GreenLight Laser in Simple Renal Cyst

**DOI:** 10.12669/pjms.36.7.2322

**Published:** 2020

**Authors:** Wen-zeng Yang, Yun-fei Sun, Zhen-yu Cui, Tao Ma

**Affiliations:** 1 Wen-zeng Yang, Department of Urology, Affiliated Hospital of Hebei University, Baoding, Hebei, 071030, P. R. China; 2 Yun-fei Sun, Medical College of Hebei University, Baoding, Hebei 071000, P. R. China; 3 Zhen-yu Cui, Department of Urology, Affiliated Hospital of Hebei University, Baoding, Hebei, 071030, P. R. China; 4 Tao Ma, Department of Urology, Affiliated Hospital of Hebei University, Baoding, Hebei, 071030, P. R. China

**Keywords:** Green Light Laser, Percutaneous Nephroscopy, Renal Cyst, Single-port

## Abstract

**Objective:**

To explore the therapeutic effect of percutaneous nephroscopy combined with Green Light laser on simple renal cyst.

**Methods:**

A retrospective analysis was conducted to review the clinical data, surgical procedures, intraoperative bleeding, postoperative adverse reactions, and length of stay of 32 patients who had been admitted to Affiliated Hospital of Hebei University from January 2018 to February 2019. All patients had been diagnosed with simple renal cyst by imaging examination and met the surgical indications for single-port percutaneous nephroscopy combined with GreenLight laser for unroofing and decompression of the renal cyst. Among the 32 patients, there were 18 males and 14 females, with 15 cases on the left and 17 on the right. The patients aged 38 to 62 years old, with an average of 45 years old. Thirteen cases were hospitalized mainly due to complaint of lumbar pain, and 19 cases were admitted after a renal cyst was found by physical examination. The diameter of the cyst ranged from 4.2 to 9.1 cm, with an average of 6.1 cm. A percutaneous nephroscopic channel was established during the surgery. Once a nephroscope was placed into the cyst, GreenLight laser (energy of 80W) was used to remove the free cyst wall 0.3cm from the renal parenchymal margin under direct vision. After the incision margin was observed with no obvious exudation under microscope, the cyst wall was removed through the channel and sent for pathological examination. A drainage catheter was placed near the cyst cavity.

**Results:**

All the 32 patients were successfully operated, without transition to laparoscopic and open surgery. The operations took 30 to 62 minutes, with an average of 45 minutes. The intraoperative bleeding ranged from three to 14 ml, with an average of 10 ml. No adverse events such as postoperative infection, fever, or active bleeding occurred. The drainage catheters were removed one to three days after operation, with an average of 1.5 days after operation. The drainage volume was 20 to 55 ml, with an average of 35 ml. No obvious liquid extravasation was seen in all cases. The length of stay after operation ranged from three to five days, with an average of 3.5 days. Postoperative pathological reports all suggested renal cyst wall. The patients were followed up for six months, and no cyst recurred.

**Conclusions:**

Single-port percutaneous nephroscopy combined with Green Light laser could provide significant clinical effect in treating simple renal cyst with minimal trauma.

## INTRODUCTION

Simple renal cyst is the most common benign kidney disease. Studies have shown that the condition can be developed from the tubular diverticulum, with the highest incidence among kidney cystic diseases. It is commonly found in adults over 50 years old, with an incidence rate of approximately 33% in those over 60 years old, but it is rare in children. The incidence rate increases with age.^1^,^2^ Changes in kidney function and morphology after the onset can cause renal insufficiency, and severe cases can result in renal failure. For cases associated with lumbar pain or a simple renal cyst of more than four cm in diameter, surgical intervention is required. Currently, the mainstream surgical approach is laparoscopy for unroofing and decompression of renal cyst.^3^,^4^ In this paper, the 32 patients with simple renal cyst admitted to our hospital were investigated as subjects, in an attempt to evaluate the clinical efficacy and safety of single-port percutaneous nephroscopy combined with GreenLight laser for unroofing and decompression in the treatment of simple renal cyst.

## METHODS

This is a retrospective study. A total of 32 patients with simple renal cyst who had been admitted to Affiliated Hospital of Hebei University from January 2018 to February 2019 were enrolled in this study. All patients underwent single-port percutaneous nephroscopy combined with GreenLight laser for unroofing and decompression. Among the 32 patients, 13 cases were hospitalized mainly due to complaint of lumbar pain, and 19 cases were admitted after a renal cyst was found by physical examination. There were 18 males and 14 females, aged 38 to 62 years old, with an average of (48.5±10.8) years old. All cases were diagnosed as simple renal cyst (dorsal) upon admission, which ranged from 4.2 to 9.1 cm in diameter, with an average of 6.1cm. Among them, 15 cases were found on the left and 17 on the right. Twelve patients had complications with hypertension, which was adjusted with antihypertensive medication before operation, and the blood pressure was lowered and controlled. Eight patients had complications with diabetes, hence insulin should be administered throughout the perioperative periods to stabilize the blood glucose level. One patient was found with urinary tract infection through routine urine test, the urine culture and drug sensitivity should be confirmed, the use of sensitive antibiotics should be guided before operation, and then the operation could be scheduled after review of urine routine became normal.

### Ethical approval

The study was approved by the Institutional Ethics Committee of Affiliated Hospital of Hebei University at January 30, 2020, and written informed consent was obtained from all participants.

### Inclusion criteria

**Fig.1:**
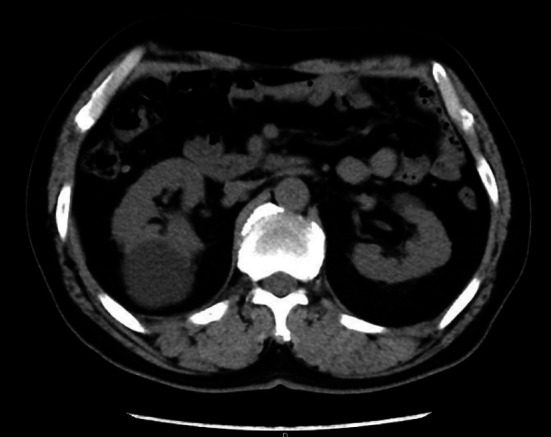
CT plain scan.

**Fig.2:**
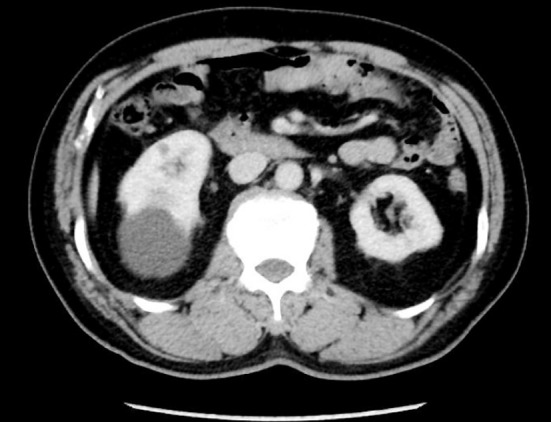
Enhanced CT.

**Fig.3:**
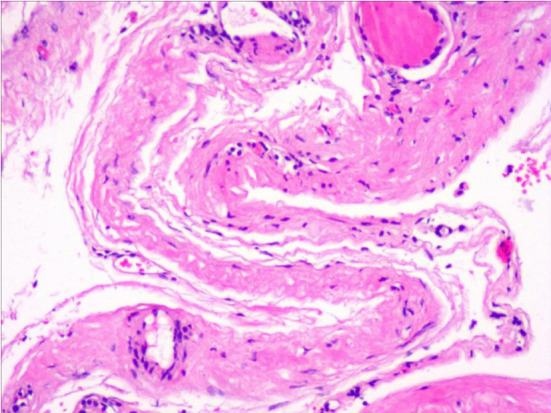
Postoperative pathological diagnosis.

a.) Cystic space-occupying lesion located on the dorsal side suggested by urinary Color Doppler ultrasound, b.) A cyst of more than >4 cm in diameter confirmed by CT plain scan, with cystic renal tumor excluded by enhanced scan. Figs. 1, 2, 3.) The cyst cavity not communicating with the collecting duct system of the kidney as suggested by intravenous pyelography.

### Exclusion criteria

a.) Polycystic kidney or cystic renal tumor, b.) Complication with other serious organic lesions such as bleeding diseases, cerebral infarction, and myocardial infarction, c.) Any surgical contraindication found by preoperative examination.

All patients followed the surgical requirements: General anesthesia was administered. After successful anesthesia, the patient was placed in a side-lying position (on either side), and the lumbar portion should be heightened with the pillow. Then the patient was routinely disinfected and covered with sterile drapes, and the light source and flushing system were powered on: 1) A percutaneous nephroscopy channel was established. Once a suitable puncture point was selected under ultrasound guidance, a puncture trocar was inserted into the cyst, and then the needle core was withdrawn. A guide wire was placed after the cystic fluid flowed out. After the needle passage was dilated with a fascial dilator along the guide wire, a balloon catheter was inserted along the wire. Then the catheter was connected to a pneumatic pump, and the pressure was increased by water injection to 25 KPa for three minutes. After the sheath was inserted, the balloon dilator (F30) was withdrawn and the nephroscope was placed. 2) After the nephroscope was placed into the cyst, the cyst wall could be observed to be smooth under microscope, so that diseases such as cystic kidney cancer could be excluded. GreenLight laser (energy of 80W) was used to remove the free cyst wall 0.3cm from the renal parenchymal margin under direct vision. 3) After the incision margin was observed with no obvious exudation under microscope, the cyst wall was removed through the channel and sent for pathological examination. A drainage catheter was placed near the cyst cavity.

Surgical procedures, intraoperative bleeding, postoperative adverse reactions, length of stay, drainage catheter removal time and drainage volume, liquid extravasation, and relapse were noted during follow-up.

## RESULTS

All the 32 patients were Successfully operated, without transition to laparoscopic and open surgery. The operations took 30 to 62 minutes, with an average of 45 minutes. The intraoperative bleeding ranged from three to 14 ml, with an average of 10 ml. No adverse events such as postoperative infection, fever, or active bleeding occurred. The drainage catheters were removed one to three days after operation, with an average of 1.5 days after operation. The drainage volume was 20 to 55 ml, with an average of 35 ml. No obvious liquid extravasation was seen in all cases. The length of stay after operation ranged from three to five days, with an average of 3.5 days. Postoperative pathological reports all suggested renal cyst wall. Fig.3 The patients were followed up for six months, and no cyst recurred.

## DISCUSSION

As annual physical examinations become widely adopted, the diagnosis rate of simple renal cyst has gradually increased. However, since simple renal cyst progresses slowly and often has no significant effect on renal function, the condition is usually treated conservatively. Clinically, a cyst of less than four cm in diameter can be regularly followed up, to observe the changes in its size, morphology, and internal texture.^5^ For cases associated with lumbar pain, hematuria, or a simple renal cyst of more than four cm in diameter, surgical treatment is needed to minimize the chance of misdiagnosis and the continuous damage to renal functions.^6^ There are many surgical methods for clinical treatment of renal cyst. Puncture and drainage can be performed under ultrasound guidance.^7^ Although it is minimally invasive, this approach leads to a high recurrence rate up to 30% to 78%,^8^ and two or even more surgeries may be needed.^9^ Moreover, patients with lesions located in particular sites should not be punctured. A sclerosing agent can also be injected into the cyst, but it may be absorbed to affect the renal parenchyma, and there is a risk of spillage. Although conventional open surgery can achieve the purpose of treatment, it brings great damage to the body, leading to slow recovery and multiple complications.^10^,^11^ With the development of minimally invasive techniques, laparoscopic unroofing and decompression of renal cyst is currently the preferred option for the surgical management. The endogenous and parapelvic cysts can significantly benefit from the advantages of flexible ureteroscopy.^12^,^13^ In this paper, nevertheless, only the simple renal cyst on the dorsal side would be discussed.

The single-port percutaneous nephroscopy features advantages in the following aspects:

### Channeling

It requires only one operation channel, and the use of ultrasound guidance during the operation will be beneficial to the locating of the cyst, and the puncture can be made easier,^14^ leaving minimal trauma to the body and almost no bleeding. The multi-port laparoscopic approach, however, requires the manual establishment of three operating channels and retroperitoneal space, which causes a high risk of renal vascular injury and strictly requires the surgeon’s operational proficiency.

### Patient position

The multi-port laparoscopic approach requires a supine position during surgery, but some patients cannot lie supine due to spinal deformity or medical conditions.

### Technical difficulty

It is not technically difficult, as there is no need to manually establish a retroperitoneal space, thereby reducing the occurrence of subcutaneous emphysema and acidosis. It is performed under the direct view via the nephroscope, reducing the chance of vascular injury and providing a clear observation of the boundary between the renal cyst wall and the renal parenchyma. Therefore, it would be safer to operate.

### Complications

It omits the dissection of the perirenal space and fat during surgery, allows less postoperative exudation, and enables a shorter removal time of the drainage catheter after surgery. Proficiency in surgical techniques is key to improving the success rate of surgery and reducing perioperative complications.

Green-Light laser has obvious advantages in vaporization. Its penetration depth into the tissue is 800 nm, and extremely high laser energy can be concentrated on the superficial tissue level. As a result, a higher power per unit volume of the local tissue, which allows the tissue to evaporate more efficiently. The GreenLight laser is used to completely destroy the integrity of the cyst wall during the operation, completely remove the cyst wall protruding from the renal parenchyma, and completely evaporate the cyst wall that cannot be removed and borders with the renal parenchyma. By destroying its function, the residual cyst wall will cause a lower chance of recurrence.

The effect of F30 is better than that of F24. During the operation, a larger operating channel can significantly increase the gap between the nephroscope and the sheath, so as to increase the water output speed and make the surgical field clearer, which will facilitate the return of water. By doing so, the postoperative infections can be avoided, and there will be no or significantly less postoperative extravasation than using F24. Meanwhile, a larger gap makes the scope’s movement and angle less restricted. This shortens the operation time to a certain extent and thus reduces the risk of surgery.

Although single-port percutaneous nephroscopy has the above advantages in treating renal cyst, this technique is not suitable for all types of renal cyst patients. The minimal trauma is achieved by reduced number of operating channel, and only one operation tool can be used. For complex renal cyst, laparoscopy and percutaneous nephroscopy should be selected carefully to ensure smooth surgery and patient safety.

### Limitations of the study

This study is a retrospective study. In order to compare laparoscopic renal cyst unroofing with other laser treatments, prospective randomized controlled trials should be further improved.

## CONCLUSION

Single-port percutaneous nephroscopy combined with Green Light laser for unroofing and decompression has good clinical effects and high safety, and this technique should be widely applied. However, this study has limitation of an insufficient sample size, hence further studies should be conducted in depth with larger sample size, with interference factors removed, in order to obtain more reliable and objective results.

### Authors’ Contributions:

**WZY and ZYC** designed this study and prepared this manuscript.

**ZYC is** responsible and accountable for the accuracy or integrity of the work.

**TM** collected and analyzed clinical data.

**YFS** significantly revised this manuscript.
